# *Paenibacillus* sp. Strain OL15 Immobilized in Agar as a Potential Bioremediator for Waste Lubricating Oil-Contaminated Soils and Insights into Soil Bacterial Communities Affected by Inoculations of the Strain and Environmental Factors

**DOI:** 10.3390/biology11050727

**Published:** 2022-05-09

**Authors:** Neelawan Pongsilp, Pongrawee Nimnoi

**Affiliations:** 1 Department of Microbiology, Faculty of Science, Silpakorn University, Nakhon Pathom 73000, Thailand; pongsilp_n@su.ac.th; 2 Department of Microbiology, Faculty of Liberal Arts and Science, Kasetsart University, Nakhon Pathom 73140, Thailand

**Keywords:** biodegradation, cell immobilization, bacterial community, bacterial diversity

## Abstract

**Simple Summary:**

Waste lubricating oil contamination is a global environmental problem. This study demonstrates the potential of using agar as an immobilization material to maintain the survival and establishment of the bioremediator. We found that the *Paenibacillus* sp. strain OL15 was a promising bacterial strain which tolerated high concentrations of waste lubricating oil and exhibited high degradation ability. The inoculations of the strain increased total bacterial diversity in the oil-contaminated soils. Soil pH, electrical conductivity (EC), organic matter (OM), nitrogen, potassium, manganese, iron, and zinc were the factors determining the fertility of the oil-contaminated soil environments. Our work develops and implements an efficient bioremediation system employing the promising bioremediator and immobilization procedure for degradation of waste lubricating oil in the soil environments.

**Abstract:**

Waste lubricating oil is a widespread common soil pollutant. In this study, the waste lubricating oil degraders were isolated from the oil-contaminated soil. The bacterial strains OL6, OL15, and OL8, which tolerated a high concentration (10%) of waste lubricating oil, presented the degradation efficiency values (measured in culture broth) of 15.6 ± 0.6%, 15.5 ± 1%, and 14.8 ± 1%, respectively, and belonged to the genera *Enterobacter*, *Paenibacillus*, and *Klebsiella*, respectively. To maintain long survival, immobilization of a promising bioremediator, *Paenibacillus* sp. strain OL15, in agar exhibited the significantly highest number of surviving cells at the end of a 30-day incubation period, as compared to those in alginate and free cells. Remarkably, after being introduced into the soil contaminated with 10% waste lubricating oil, the strain OL15 immobilized in agar conferred the highest degradation percentage up to 45 ± 3%. Due to its merit as a promising soil pollutant degrader, we investigated the effect of an introduction of the strain OL15 on the alterations of a bacterial community in the oil-contaminated soil environments using 16S rRNA amplicon sequencing. The result revealed that the *Proteobacteria*, *Acidobacteriota*, *Firmicutes*, and *Actinobacteriota* were predominant phyla. The introduction of the strain affected the soil bacterial community structures by increasing total bacterial diversity and richness. The proportions of the genera *Pseudomonas*, *Vibrio*, *Herbaspirillum*, *Pseudoalteromonas*, *Massilia*, *Duganella*, *Bacillus*, *Gordonia*, and *Sulfurospirillum* were altered in response to the strain establishment. Soil pH, EC, OM, total N, P, Mg, Fe, and Zn were the major factors influencing the bacterial community compositions in the oil-contaminated soils.

## 1. Introduction

Waste or used lubricating oil is rapidly increasing every year due to a global increase in transportation and high consumption of petroleum products [1,2]. Most lubricating oil generally consists of chemical additives and base fluid that are a complex mixture of heavy metals, polycyclic aromatic hydrocarbons (PAHs), and chlorinated hydrocarbons (CHCs). These agents are chronic hazards contributing to neurotoxic effects, carcinogenicity, and mutagenicity in a biological system [3,4]. In spite of the toxicity of lubricating oil, adequate attention has not been given to its proper disposal [5]. Contamination of soil by waste lubricating oil has become a severe problem in developing countries around the world where policies on the environmental regulation are likely to be insufficient [6]. Such contamination directly and indirectly adulterates soil fertility by decreasing soil organic matter content and the availability of nutrients, oxygen, and water; consequently causing an imbalance of soil microbiome that plays an important role in soil nutrient recycling [4,7].

Although waste lubricating oil can be re-refined to lube base oil, the recycling process is high cost and time-consuming because of the high contamination of waste oil and variation among formulas of different manufacturers [3]. To cope with this problem, microbial bioremediation is an attractive method for the removal of hydrocarbon pollutants nowadays because of its cost-effectiveness, simple operation, and environmental friendliness [8,9]. Bioremediation is a process of using living microorganisms to degrade and detoxify target pollutants. The two approaches most widely used are bioaugmentation, in which a culture of exogenous or native microorganism is introduced into a contaminated site in order to enhance the microbial number or replace native microorganisms [10], and biostimulation, in which a large amount of nutrients is supplied into a contaminated site in order to stimulate the growth of native microorganisms [11]. In previous reports, bacteria which were isolated from the contaminated fields and had the ability to degrade waste lubricating oil, belonged to the genera *Pseudomonas*, *Burkholderia*, *Rhodococcus*, *Achromobacter*, *Bacillus*, *Arthobacter*, *Acinetobacter*, *Sphingomonas*, *Ochrobactrum*, and other related ones [3,8]. Many studies recommended the use of a mixed bacterial culture or a consortium in bioaugmentation for enhancing the degradation rate and providing effective digestion of a wide range of pollutants [9,12]. Thus, it merits an effort to screen the promising strains with high ability that are significantly effective in degrading waste lubricating oil.

Indeed, there are still some problems regarding the survival and establishment of the inoculants in bioaugmentation techniques. Previous research reported that the inoculants were unable to compete with soil predators and native microorganisms in establishing niches for their survival; consequently leading to short-period degradation [3,11,13]. Thus, immobilization is likely to be an efficient alternative technique for the protection of the inoculants against soil predators and natural competition with soil microbiomes. Wang et al. [13] succeeded in using alginate and alginate–diatomite as immobilization materials to support the growth and enhance the degradation ability of the inoculants in the oil-contaminated soil. Nimnoi et al. [14]; and Pongsilp and Nimnoi [15] demonstrated the potential of using agar as an immobilization material to prolong the survival time and support the establishment of the inoculant in the competitive soil environments. Since there is no report on using agar as an immobilization material for bioaugmentation purposes to date, we therefore undertook the present study to isolate the efficient waste lubricating oil degrader for use as a bioremediator and to evaluate the potential of using agar as an immobilization material, compared with that of alginate and free cells for supporting the niche establishment and enhancing the degradation ability of the bioremediator in the soils highly contaminated with waste lubricating oil. Moreover, since information on the effect of the inoculant on the bacterial community in waste lubricating oil-polluted soil is currently woefully inadequate, this study also aims to determine the effect of the bioremediator on the shift in bacterial community structure which responds to environmental factors in the soil environments highly polluted by waste lubricating oil. This study presents the new considerable insights into the bacterial community structures during bioremediation of the highly polluted soil environments.

## 2. Materials and Methods

### 2.1. Sampling of Soil Contaminated with Waste Lubricating Oil

Soil contaminated with waste lubricating oil was collected at 0–20 cm depth from an automobile repair shop in the Mueang district, Lopburi province of Thailand, stored in sterile plastic bags, and transported to the laboratory within 24 h for isolation of bacteria degrading waste lubricating oil.

### 2.2. Isolation of Bacteria Degrading Waste Lubricating Oil

Ten grams of the oil-contaminated soil was transferred to 150 mL of mineral salt medium (MSM) (HiMedia, Maharashtra, India) containing 2% (v v^−1^) waste lubricating oil and incubated in a rotary shaker incubator at 37 °C with shaking at 120 rpm for 7 days. In the experiments, waste lubricating oil was autoclaved at 121 °C and 15 psi for 15 min prior to being aseptically added into sterile MSM. At the end of a 7-day cultivation, 10 mL of the bacterial culture from the first step was added into 150 mL of MSM containing 2% (v v^−1^) waste lubricating oil and further incubated in the same condition as described above in order to selectively enrich bacteria degrading waste lubricating oil. Then, the bacterial culture from the second step was diluted by using a 10-fold-dilution technique. Appropriate dilutions were spread onto nutrient agar (NA) (Merck, Darmstadt, Germany) containing 2% (v v^−1^) waste lubricating oil and incubated at 37 °C for 5 days. Then, colonies showing different morphological characteristics were selected. The selected isolates were maintained in NA slant cultures by chilling at 4 °C for short-term preservation and in 20% glycerol at -20 °C for long-term preservation.

### 2.3. Screening Test for Effective Waste Lubricating Oil Degraders

In total, 1 ml of a fresh overnight bacterial culture of each isolate (optical density at 600 nm of 1.00) was transferred to 150 mL of MSM containing waste lubricating oil at four different concentrations [3%, 5%, 7%, and 10% (v v^−1^)]. Each subculture was incubated in a rotary shaker incubator at 37 °C with shaking at 120 rpm for 7 days. The remaining waste lubricating oil after bacterial cultivation was quantitatively determined. Waste lubricating oil was extracted twice from culture supernatant by using 200 mL of hexane. The solvent was evaporated to dryness by a vacuum rotary evaporator, then the dry weight was determined and the oil degradability was evaluated based on the weight loss method described by Shirai et al. [16].

### 2.4. 16S rRNA Gene Sequence Analysis of the Selected Bacterial Strains Capable of Degrading Waste Lubricating Oil

The top three most effective isolates with the ability to degrade waste lubricating oil were selected. Each isolate was propagated in MSM containing 2% (v v^−1^) waste lubricating oil and incubated at 37 °C with shaking at 120 rpm for 5 days. The cell pellet was obtained from the mid-log-phase culture by centrifugation at 12,000 rpm for 5 min and washed twice with nuclease-free water (Invitrogen, Waltham, MA, USA). The genomic DNA of the selected isolates was extracted by using a GF-1 bacterial DNA extraction kit (Vivantis, Selangor, Malaysia). The 16S rRNA gene of the selected isolates was amplified by using Quick Taq HS DyeMix (Toyobo, Osaka city, Japan) and a set of universal primers, 8F and 1045R [17]. The polymerase chain reaction (PCR) mixture and condition were employed as described by Nimnoi et al. [14]. The amplified 16S rRNA gene fragments (approximately 1 kb in size) were purified by using a GF-1 AmbiClean kit (Vivantis, Selangor, Malaysia). The purified PCR products were directly sequenced at the 1st Base Inc. (Wilayah Persekutuan, Malaysia). The 16S rRNA gene sequences were compared with known nucleotide sequences in a database of the National Center for Biotechnology Information (NCBI, Bethesda, MD, USA; http://www.ncbi.nlm.nih.gov (accessed on 4 March 2022)) by using the BLASTN program. Phylogenetic tree was constructed by using the MEGA X software [18].

### 2.5. Cell Immobilization and Determination of Bacterial Survival

Three selected bacterial strains were immobilized in agar and alginate according to the immobilization procedure described by Nimnoi et al. [14] to obtain the initial cell number of 10^7^ cells g^−1^ immobilization material. Then, the cell survivability, after being immobilized in agar and alginate and then stored at 37 °C for 15 and 30 days, compared with free cells in sterile normal saline, was determined. Ten grams of each agar cube and alginate bead containing immobilized cells was added into 90 mL of sterile normal saline and incubated in a rotary shaker incubator at 37 °C with shaking at 120 rpm for 1 h. Then, the released cell number was determined on NA plates by the standard plate count method.

### 2.6. Degradation Ability of Immobilized and Free Cells as Estimated in the Soil Contaminated with Waste Lubricating Oil

The degradation ability of the top three most effective isolates with the ability to degrade waste lubricating oil, after being immobilized in agar and alginate and introduced into the oil-contaminated soil, was evaluated, as compared with that of bacterial cell suspensions. In order to control the concentration and homogeneity of the oil contamination in soil, the initial soil was artificially contaminated with 10% (v v^−1^) waste lubricating oil and mixed thoroughly for 1 h. Then, bacterial cells immobilized in agar and alginate and cell suspensions were inoculated into pots containing the oil-contaminated soil to reach a final concentration of 10^9^ cells kg^−1^ soil. The oil-contaminated soil without inoculation was set as a control. A total of 30 pots (three replications of each treatment including each strain immobilized in agar and alginate and a control) were undertaken and arranged in a randomized block design, incubated in a greenhouse, and watered with sterile water at 500 mL kg^−1^ soil at 7-day intervals throughout an entire 30-day period. At the end of an incubation period, the soil of each treatment was collected and the oil degradability was determined by the weight loss method [16]. The strain exhibiting the highest value of oil degradability was selected and subjected to soil physicochemical analyses, soil DNA extraction, and Illumina next-generation sequencing (NGS).

### 2.7. Soil Physicochemical Analyses

The most effective strain was used as a bioremediator in setting up four treatments: the uninoculated oil-contaminated soil as a control (treatment A); the oil-contaminated soil inoculated with the agar-immobilized strain (treatment B); the oil-contaminated soil inoculated with the alginate-immobilized strain (treatment C); and the oil-contaminated soil inoculated with a cell suspension of the strain (treatment D). In total, 2 kg of the soil from each treatment was air-dried, sieved through a sterile 10-mesh laboratory sieve with a 2-mm aperture size, and evaluated for soil physicochemical properties. The electrical conductivity (EC) and pH were measured as described by Jackson [19]. Organic matter (OM) content was determined following the method of Walkley and Black [20]. Amounts of P, Fe, Ca, and Zn were determined by the Bray II method [21]. Flame photometric method [19] was used to analyze amounts of K and Mg. The quantity of N was analyzed as described by Li et al. [22].

### 2.8. Soil DNA Extraction and Illumina NGS

Soil DNA extraction was performed with the soil samples from three replicates of each treatment by using a NucleoSpin soil kit (Macherey-Nagel, Duren, Germany) following the manufacturer’s instruction. Then, the quantity and purity of extracted DNA were evaluated by using a DS-11FX+ Spectro/Fluorometer (DeNovix, Wilmington, DE, USA). PCR amplification of V4 variable region of the 16S rRNA gene was conducted with a pair of primers, 515F and 806R, linked to the barcodes [23,24] and Phusion High-Fidelity PCR Master Mix (New England Biolabs, Ipswich, MA, USA). The amplified DNA libraries were constructed with a TruSeq DNA PCR-Free sample preparation kit (Illumina, San Diego, CA, USA) and analyzed with a HiSeq2500 PE250 sequencing platform (Illumina, San Diego, CA, USA) according to the manufacturer’s instructions. Negative controls (reactions with sterile water) were carried out through amplification and sequencing. Data were converted to fastq files.

### 2.9. Data Processing and Bioinformatic Analyses

Sequence reads were merged by the FLASH software version 1.2.7 [25]. Quality filtering of the raw tags was performed by using the QIIME software version 1.7.0 [26] in order to select the high-quality clean tags. The UCHIME algorithm was used for detection of chimera sequences by comparison of the obtained tags and reference sequences in a database. Then, the final effective tags were acquired after removal of chimera sequences [27]. For operational taxonomic unit (OTU) clustering and species annotation, all effective tags were bioinformatically processed with the Uparse software version 7.0.1001 [28]. Sequences with ≥97% similarity were assigned to the same OTUs. The Mothur software version 1.36.1 [29] was used to align each representative sequence with known sequences from the SSU rRNA database of SILVA for species annotation at each taxonomic level [30]. The MUSCLE program version 3.8.31 was used to analyze the phylogenetic relationship of all OTUs derived from representative sequences [31].

### 2.10. Statistical Analyses

The QIIME software version 1.7.0 was used to calculate the parameters regarding alpha diversity, including community diversity (Shannon–Weaver and Simpson’s indices), community richness (Chao1 and ACE estimators), and index of sequencing depth (the Good’s coverage). The analyzed data were displayed by the R software version 2.15.3 [32]. Principal coordinate analysis (PCoA) was performed to visualize complex, multidimensional data, which were then displayed by the WGCNA, stat, and ggplot2 packages [33] in the R software version 2.15.3. The unweighted-pair group method with arithmetic mean (UPGMA) clustering, which was performed as a type of hierarchical clustering method to interpret the distance matrix using average linkage, was conducted by using the QIIME software version 1.7.0. Linear discriminant analysis (LDA) effect size (LEfSe) analysis that is available in the LEfSe software version 1.1.0 [34] was employed to identify differentially abundant groups among samples. The nonparametic method, analysis of molecular variance (AMOVA), was calculated by using the Mothur software version 1.36.1. Analysis of similarity (ANOSIM) was computed by the R software version 2.15.3 to determine whether the bacterial community structures have significant inter- and inner-group differences. The canonical-correlation analysis (CCA) was analyzed by using the PAST software version 4.03 [35]. The oil degradability based on the weight loss method, soil physicochemical parameters, and alpha diversity indices were subjected to analysis of variance (ANOVA) using Tukey’s test. The association of soil physicochemical parameters with bacterial communities was analyzed by Spearman’s correlation. ANOVA and Spearman’s correlation were performed with the SPSS software version 19.0 (IBM Corp., Armonk, NY, USA). All data analyses were performed with triplicate samples. The p-value was considered indicative of significance if it was equal to or less than 0.05.

## 3. Results

### 3.1. Isolation and Screening Test of Effective Waste Lubricating Oil Degraders

A total of 15 bacterial isolates, which were selected and purified from the soil contaminated with waste lubricating oil, were subjected to determination of waste lubricating oil degradability at concentrations of 3%, 5%, 7%, and 10% (v v^−1^). As presented in Table 1, the results showed that all isolates were able to degrade waste lubricating oil at a concentration of 3% with the percentages of degradation ranging between 4.5 ± 0.5 and 38 ± 1%. The isolates OL2, OL8, and OL15 conferred the highest values, which were not significantly different from each other. At a concentration of 5%, nine isolates retained their degradability; the percentages of degradation ranged between 5 ± 4.4 and 37 ± 3%. At a concentration of 7%, seven isolates retained their degradation ability, although the percentages of degradation were decreased to between 7.7 ± 0 and 17.6 ± 0.5%. The isolates OL2 and OL15 exhibited the significantly highest values with concentrations of 5% and 7%, respectively. At a concentration of 10%, seven strains retained their degradation ability with the percentages of degradation ranging between 5.8 ± 0.6 and 15.6 ± 0.6%. The top three most effective isolates were selected based on the highest percentages of degradation at a concentration of 10%. The selected isolates included OL6 (15.6 ± 0.6%), OL15 (15.5 ± 1%), and OL8 (14.8 ± 1%), whose values were not significantly different from each other, but significantly different from those of the remaining isolates.

### 3.2. Identification of the Selected Bacterial Isolates

The bacterial isolates OL6, OL15, and OL8, which were the top three isolates capable of degrading waste lubricating oil at a concentration of 10%, were selected and identified by a comparative sequence analysis of the 16S rRNA gene. The results from the BLASTN showed that the isolates OL6, OL15, and OL8 were genetically closest to members of the genera *Enterobacter* (a maximum identity of 99%), *Paenibacillus* (a maximum identity of 99%), and *Klebsiella* (a maximum identity of 100%), respectively. To assure genus identification, the phylogenetic analysis was performed to align the 16S rRNA gene sequences of these isolates with the closest sequences of type strains retrieved from the NCBI database. The constructed phylogenetic tree (Supplementary Figure S1) diverges to three different clades. The isolate OL6 was grouped within the *Enterobacter* clade and closest to *E. tabaci* strain KCTC 42694. The isolate OL15 was clustered into the *Paenibacillus* clade and closest to *P. timonensis* strain DSM 16943. The isolate OL8 was positioned within the *Klebsiella* clade and closest to *K. quasipneumoniae* strain DSM 28211. The phylogenetic positions of these isolates strongly supported their genus identification relying on the 16S rRNA gene sequence identity. As their distinguishable genetic data from both methodologies provided useful evidence, these isolates were consequently assigned to individual strains, namely *Enterobacter* sp. strain OL6, *Klebsiella* sp. strain OL8, and *Paenibacillus* sp. strain OL15. The 16S rRNA gene sequences of these strains can be retrieved from the NCBI database with accession numbers OM761074-OM761076.

### 3.3. Survival of Immobilized Cells of the Selected Strains

The survival of the strains OL6, OL8, and OL15 immobilized in agar and alginate, after storage at 37 °C for 15 and 30 days, was evaluated and shown in Table 2. At day 15 of immobilization in agar, *Paenibacillus* sp. strain OL15 exhibited the highest number of surviving cells, followed by *Klebsiella* sp. strain OL8, and *Enterobacter* sp. strain OL6, respectively. At day 30 of immobilization in agar, the strain OL15 still exhibited the highest number of surviving cells, which was significantly different from those of other strains. When comparing the survival of the strains immobilized in alginate, at day 15, even though the strain OL15 had the highest number of surviving cells, it was insignificantly different from those of other strains. However, at day 30, *Paenibacillus* sp. strain OL15 still had the greatest value of survivability, which was significantly different from those of *Klebsiella* sp. OL8 and *Enterobacter* sp. OL6. The surviving cell numbers of the strain OL15, which was freely grown in sterile normal saline at days 15 and 30, were significantly higher than those of other strains. The surviving cell numbers of free cells in sterile normal saline were lower than those of immobilized cells for all strains. Due to the survival performances of these strains immobilized in both materials, they merit further analysis of the ability of immobilized cells to degrade waste lubricating oil contaminated in the soil environments.

### 3.4. Degradation Ability of Immobilized and Free Cells of the Selected Strains in the Soil Contaminated with Waste Lubricating Oil

The degradation ability of the selected strains immobilized in agar and alginate, after introduction into the 10% oil-contaminated soil for 30 days, was determined as compared to that of inoculations, with cell suspensions. The percentages of degradation are shown in Table 3. All inoculation treatments had higher percentages of degradation, which were significantly different from that of an uninoculated control (7 ± 1%). When using agar as an immobilization material, *Paenibacillus* sp. strain OL15 conferred the significantly highest percentage of degradation (45 ± 3%), followed by *Klebsiella* sp. OL8 (28 ± 2%), and *Enterobacter* sp. OL6 (24 ± 7%). A similar trend was observed when using alginate as an immobilization material, the strain OL15 conferred the significantly highest percentage of degradation (39 ± 1%), followed by the strains OL8 (28 ± 2%), and OL6 (14 ± 4%).

In addition, the degradation ability of *Paenibacillus* sp. strain OL15 was compared among inoculation types: immobilization in agar was still the best method providing the significantly highest percentage of degradation, followed by immobilization in alginate, and bacterial cell suspension, respectively. When comparing among strains, *Paenibacillus* sp. strain OL15 immobilized in agar and alginate showed both the significantly highest numbers of surviving cells and waste lubricating oil degradation ability, as estimated in the soil environments in a 30-day period, and therefore was selected for investigation of its effect on the distribution of soil bacterial communities in response to the change in soil physicochemical parameters.

### 3.5. Physicochemical Parameters of the Soils from Different Inoculation Types

In this study, four treatments examined were as follows: the uninoculated oil-contaminated soil as a control (treatment A); the oil-contaminated soil inoculated with the agar-immobilized *Paenibacillus* sp. strain OL15 (treatment B); the oil-contaminated soil inoculated with the alginate-immobilized strain OL15 (treatment C); and the oil-contaminated soil inoculated with a cell suspension of the strain OL15 (treatment D). Soil physicochemical properties from four treatments are listed in Table 4. Among all treatments, pH and EC were varied in the ranges of 6.73 to 7.33 and 1.7 to 2.8, respectively. Treatment A had the highest amount of OM, which was significantly different from those of other treatments, followed by treatments D, C, and B, respectively. Treatments B and C contained the highest amounts of total N, which were significantly different from those of other treatments. Treatment A had the lowest amount of total P, which was significantly different from those of other treatments. In contrast, treatment A had the highest amounts of total Mg, Fe, and Zn, which were significantly different from those of other treatments. Treatment C had the lowest amount of total Ca. The amounts of total K were not significantly different among treatments. When compared between uninoculation and inoculation treatments of the oil-contaminated soils, an uninoculation treatment had the higher values of pH, OM, Mg, Fe, and Zn, while inoculation treatments had the higher amounts of total N and P.

### 3.6. Bacterial Diversity and Richness of the Soils from Different Inoculation Types

**Figure 1:**
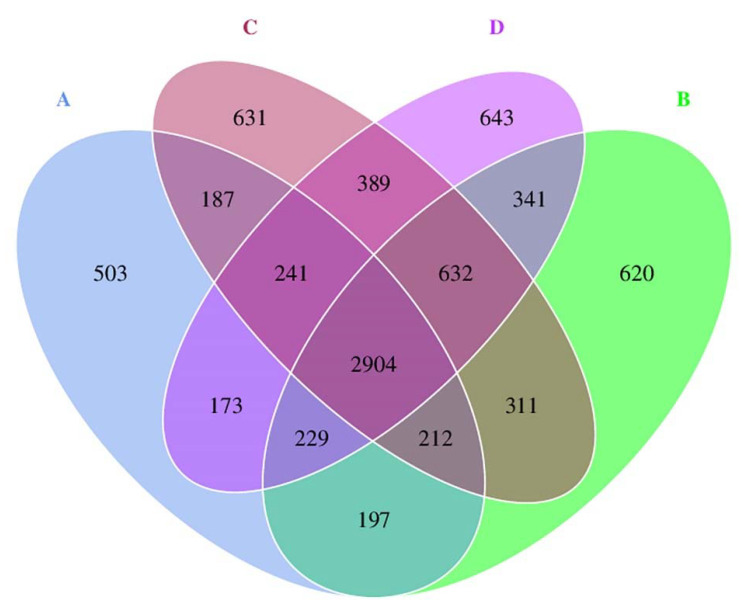
Venn diagram presenting the numbers of overlapping, unique, and shared OTUs among treatments. Treatment A, an uninoculated control; B, inoculation with *Paenibacillus* sp. strain OL15 immobilized in agar; C, inoculation with *Paenibacillus* sp. strain OL15 immobilized in alginate; D, inoculation with a cell suspension of *Paenibacillus* sp. strain OL15.

Bacterial diversity and richness of the four treatments (A-D) were revealed by Illumina NGS. A total of 1,546,560 raw reads were obtained from 12 DNA samples (three replicates/treatments). After tag merge and sequence quality control, a total of 1,523,592 qualified tags (98.51% of raw reads) were retrieved. Then, potential chimera tags were removed by the UCHIME algorithm, yielding a total of 1,166,338 taxon tags. The tags with ≥97% similarity were grouped into the same OTU. Finally, a total of 8258 OTUs were obtained from all treatments, with a mean Good’s coverage of 99%. All resulting sequences are accessible in the Sequence Read Archive of the NCBI under BioProject accession number: PRJNA809146. The Venn diagram (Figure 1) illustrated the numbers of common, overlapping, and unique OTUs between inoculated and uninoculated oil-contaminated soil environments. The diagram displayed the common 2904 OTUs among all treatments. Treatment D showed the highest unique OTUs of 643, followed by treatments C, B, and A, respectively. When comparing the common OTUs among pairs of treatments, treatments A and D shared the highest common OTUs of 3547, followed by treatments A and C (3544 OTUs), as well as treatments A and B (3542 OTUs). Moreover, the diversity indices (Shannon–Weaver and Simpson), richness indices (Chao1 and ACE), and the number of observed species in each treatment were analyzed (Table 5). Treatment D had the highest number of observed species, which was not significantly different from those of treatments C and B.

As higher values of Shannon–Weaver and Simpson indices illustrate the greater bacterial diversity, treatment A displayed the lowest bacterial diversity, which was significantly different from that of other inoculation treatments. Treatment D exhibited the highest bacterial diversity, which was not significantly different from that of treatments C and B. Treatment A also exhibited the lowest bacterial richness, as presented by the richness indices (Chao1 and ACE), which was not significantly different from that of treatment B and C. Treatment D exhibited the highest bacterial richness, which was not significantly different from that of treatments C and B. The results demonstrate that the inoculations of *Paenibacillus* sp. strain OL15 exerted the higher bacterial diversity and richness in the soils contaminated with waste lubricating oil.

### 3.7. Bacterial Community Structures of the Soils from Different Inoculation Types

**Figure 2:**
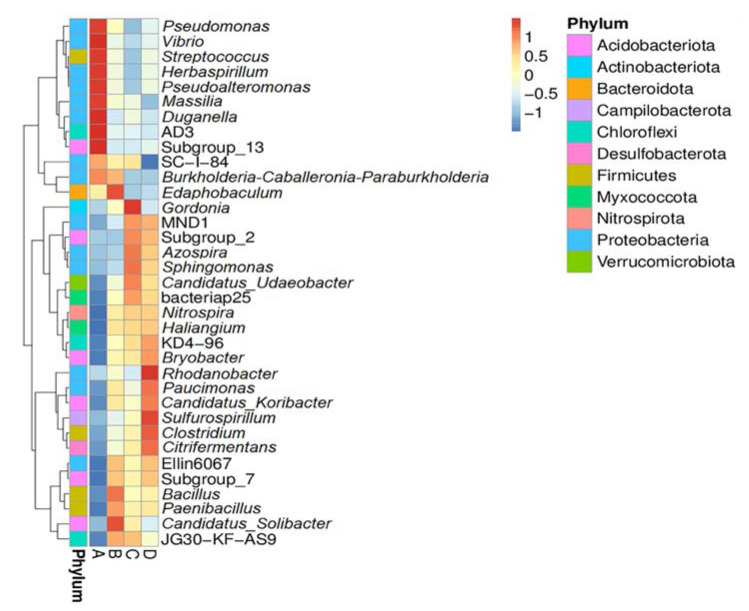
Heat map analysis of the genus distribution in each treatment. Treatment A, an uninoculated control; B, inoculation with *Paenibacillus* sp. strain OL15 immobilized in agar; C, inoculation with *Paenibacillus* sp. strain OL15 immobilized in alginate; D, inoculation with a cell suspension of *Paenibacillus* sp. strain OL15.

Among all treatments, the phylum *Proteobacteria* was mostly abundant, ranging between 55 and 71%, followed by the *Acidobacteriota* (10–11%), *Firmicutes* (2–6%), *Actinobacteriota* (1–5%), *Campilobacterota* (0.08–4%), *Chloroflexi* (2–3%), *Bacteroidota* (1–2%), *Verrucomicrobiota* (1–2%), *Desulfobacterota* (0.4–2%), and *Myxococcota* (0.9–1.7%). To determine the unique community characteristic between uninoculation and inoculation treatments, a biomarker analysis was performed using the LEfSe method (Supplementary Figure S2). The result displays different bacterial community compositions among four examined treatments. The phyla *Proteobacteria*, *Firmicutes*, *Actinobacteriota*, and *Campilobacterota* were significantly more abundant in treatments A, B, C, and D, respectively. For the finer taxonomic level, the genera *Massilia* and *Vibrio* were more common in treatment A, whereas the *Sulfurospirillum* was more common in treatment D. To further explore the distribution of each genus among all treatments, a heat map analysis was performed. The colors in a heat map chart (Figure 2) signify the abundance distribution of the bacterial community. The colors, which vary from deep blue to dark brown, indicate low- to high-levels of the relative abundance. The most abundant genera in each treatment are marked as dark brown squares in a heat map chart. In treatment A, the *Pseudomonas*, *Vibrio*, *Streptococcus*, *Herbaspirillum*, *Pseudoalteromonas*, *Massilia*, *Duganella*, AD3, and subgroup13 were the predominant genera. The *Edaphobaculum*, *Bacillus*, and *Candidatus* were more abundant than other genera in treatment B. The *Gordonia*, *Azospira*, and *Sphingomonas* were more common in treatment C. Treatment D had the *Rhodanobacter*, *Sulfurospirillum*, *Clostridium*, and *Citrifermentans* as the highly abundant genera. Remarkably, when focused on the abundance of the genus *Paenibacillus* that was used as the inoculant, the result clearly showed that treatment B (inoculation with the agar-immobilized strain) displayed the higher abundance when compared with that of treatments C (inoculation with the alginate-immobilized strain) and D (inoculation with a cell suspension). On the contrary, treatment A (an uninoculated control) displayed a deep blue color, which represented the lowest abundance of *Paenibacillus*. The proportions of the genera *Gordonia*, MND1, *Azospira*, *Sphingomonas*, *Candidatus*, bacteriap25, *Nitrospora*, *Haliangium*, KD4-96, *Bryobacter*, *Rhodanobacter*, *Paucimonas*, *Sulfurospirillum*, *Clostridium*, *Citrifermentans*, Ellin6067, Subgroup_7, *Bacillus*, *Paenibacillus*, and JG30-KF-AS9 were increased in all inoculation treatments.

**Figure 3:**
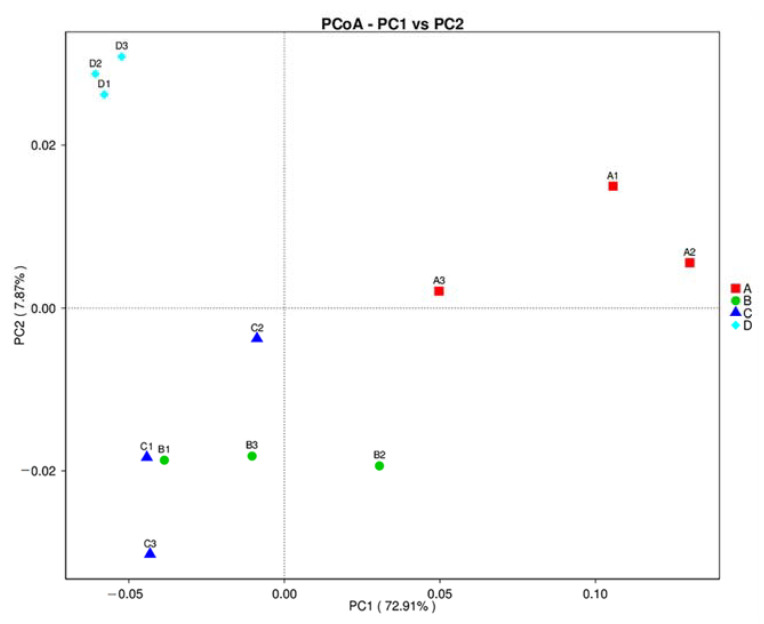
PCoA analysis of the bacterial composition similarities among treatments. Treatment A, an uninoculated control; B, inoculation with *Paenibacillus* sp. strain OL15 immobilized in agar; C, inoculation with *Paenibacillus* sp. strain OL15 immobilized in alginate; D, inoculation with a cell suspension of *Paenibacillus* sp. strain OL15.

**Figure 4:**
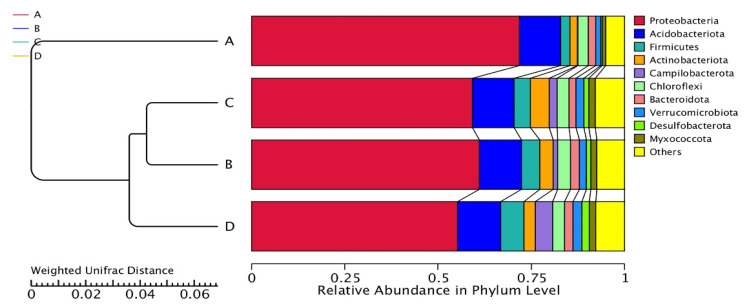
The UPGMA dendrogram of relative abundance at phylum level from treatments. Treatment A, an uninoculated control; B, inoculation with *Paenibacillus* sp. strain OL15 immobilized in agar; C, inoculation with *Paenibacillus* sp. strain OL15 immobilized in alginate; D, inoculation with a cell suspension of *Paenibacillus* sp. strain OL15.

The AMOVA method, which aimed to determine the differences in bacterial community among treatments, revealed significant variations in bacterial community structure among treatments (Fs = 7.948; *p* < 0.001). The variation analysis of inter- and inner-group bacterial community structures among treatments was evaluated by ANOSIM. The result indicates that the variations of inter-group bacterial community structure were larger than those of inner-group (*R* = 1). The multivariate variance test, which was used to estimate the significance of grouping among treatments, indicated that the bacterial community of treatment B was most similar to that of treatment C (R_2_ = 0.39). The bacterial communities of treatments A and D were most dissimilar to each other (R_2_ = 0.79). The ordination of each treatment by PCoA, depicted in Figure 3, clearly demonstrated the significant bacterial communities between treatments A and D, while the bacterial communities of treatments B and C were closest to each other. The UPGMA dendrogram of the relative abundance at the phylum level demonstrated the relation among treatments (Figure 4). The result showed that treatments B and C were closest to each other and linked with treatment D, whereas treatment A was placed apart from other treatments. These results imply the differences in bacterial community structure between an uninoculation treatment (treatment A) and inoculation treatments (treatments B, C, and D), where an uninoculation treatment had the lower bacterial diversity and richness.

### 3.8. Effect of Environmental Factors and Paenibacillus sp. Strain OL15 on the Distribution of the Soil Bacterial Communities

The effect of soil physicochemical characteristics and the inoculant, *Paenibacillus* sp. strain OL15, on the distribution of soil bacterial communities was analyzed. The results (Supplementary Table S1) showed that the gamma-*Proteobacteria* was significantly positively associated with total Mg. The alpha-*Proteobacteria* was significantly positively associated with total P and significantly negatively associated with pH and total Zn. The *Campylobacteria* was significantly negatively associated with total Mg. The *Actinobacteria* was significantly positively associated with EC, total N, and total P, and significantly negatively associated with pH, OM, total Mg, total Fe, and total Zn. Members of the *Clostridia* and *Desulfuromonadia* were significantly negatively associated with total Mg. The *Bacilli* was significantly positively associated with total P. The OTUs and numbers of observed species were significantly positively associated with total P and significantly negatively associated with total Mg. To definitively determine the relationship between bacterial community composition and environmental factors, the CCA was analyzed and the result indicates that pH, OM, total N, P, Mg, Fe, and Zn were the major factors affecting the bacterial community composition (Supplementary Figure S3). However, when comparing the top ten most abundant bacterial classes presented in each treatment (Table 6), treatment A (an uninoculated control) had the highest number of the gamma-*Proteobacteria*, which was significantly different from those in other treatments.

In contrast, the numbers of the *Campylobacteria*, *Clostridia*, and *Desulfuromonadia* in treatment A were significantly lower than those in other treatments. The numbers of the alpha-*Proteobacteria*, *Actinobacteria*, *Bacilli*, and *Verrucomicrobiae* in treatment A were lowest and significantly different from those in some other treatments. Noticeably, the number of the *Bacilli* in treatment B was significantly increased when compared with that in other treatments. In contrast with treatment A, the numbers of the *Campylobacteria*, *Clostridia*, and *Desulfuromonadia* in treatment D were significantly higher than those in other treatments. The numbers of the *Acidobacteriae* and *Bacteroidia* were not significantly different among treatments. The gamma-*Proteobacteria* was the only one class which had the highest number and was significantly abundant in an uninoculation treatment (treatment A), whereas the *Campylobacteria*, *Clostridia*, and *Desulfuromonadia* were the predominant classes, which were significantly increased in all inoculation treatments.

## 4. Discussion

Waste lubricating oil has become a serious environmental problem, especially when its contamination in soil is rapidly increasing due to high demand in the usage of petroleum products around the world [1]. Waste lubricating oil is a complex mixture of hydrocarbons and contains potentially toxic substances, such as heavy metals and PAHs, which can seep into surface and ground water; consequently resulting in lethal chronic conditions [2,6]. The use of bioremediation to eliminate waste lubricating oil in contaminated soil is a promising method that provides advantages over physical and chemical methods [13]. In this study, 15 waste lubricating oil degraders were successfully isolated. All bacterial isolates were capable of degrading waste lubricating oil at a concentration of 3%. Nine, seven, and seven isolates retained the ability to degrade waste lubricating oil at higher concentrations of 5%, 7%, and 10%, respectively. The degradation ability was correlated inversely with concentration of waste lubricating oil. The highest percentages of degradation ability at concentrations of 3%, 5%, 7%, and 10% were 38 ± 1%, 37 ± 3%, 17.6 ± 0.5%, and 15.6 ± 0.6%, respectively. These results agreed with Wang et al. [13] who reported that the degradation potential of hydrocarbon-degrading bacteria was decreased with an increase in the concentration of crude oil. Abioye et al. [1] demonstrated that the percentages of waste lubricating oil biodegradation were decreased from 92% to 55% when the concentrations of waste lubricating oil were increased from 5% to 15%. Bhattacharya et al. [3] reported that the waste lubricating oil degradation efficiencies of *Ochrobactrum* sp. C1 at concentrations of 2%, 5%, and 10% were 30.43%, 27.32%, and 18.74%, respectively. A decrease in waste lubricating oil degradation efficiency occurs when the concentration of waste lubricating oil is increased, possibly because a high concentration of hydrocarbons, which contain PAHs, heavy metals, chlorinated alkanes, and undispersed oil slicks, causes inhibition of biodegradation by limiting oxygen and bio-accessibility or emitting volatile compounds that impose toxic effects on microorganisms [1,8,9].

Moreover, in this study, we found that *Enterobacter* sp. strain OL6, *Klebsiella* sp. strain OL8, and *Paenibacillus* sp. strain OL15 were the top three most effective strains at degrading waste lubricating oil. As reported by previous research, bacteria which were isolated from hydrocarbon-contaminated soils and had the ability to degrade waste lubricating oil belonged to several genera, including *Pseudomonas*, *Paenibacillus*, *Bacillus*, *Proteus*, *Klebsiella*, *Micrococcus*, *Acinetobacter*, *Nocardia*, *Enterobacter*, *Salmonella*, and *Escherichia* [3,9,36,37,38,39]. Bacteria belonging to different genera exhibited different degrees of hydrocarbon utilization when inoculated into MSM medium containing waste lubricating oil as the sole carbon and energy source. The different rates of degradation may be because different genera possess different metabolic pathways, efficient catabolic genes, and enzymes for degradation of petroleum products [9,37]. Waste lubricating oil generally consists of several chemical additives and, especially, base fluid as the main fraction. Base fluid is a mixture of linear and branched paraffins, complex aromatic hydrocarbons, as well as cyclic alkanes [40]. In bacteria, dehydrogenases and mono- and di-oxygenases are specialized enzymes with the ability to break down complex aromatic hydrocarbons and recalcitrant compounds into lower-molecular-weight benzene derivatives, such as arene oxide, phenol, and catechol [7,9]. Shibulal et al. [39], who studied the biotransformation of heavy crude oil by *Paenibacillus ehimensis* strain BS1 in Bushnell Haas medium with 1% (w v^−1^) heavy crude oil in a 12-day period, reported that the gas chromatography-mass spectrometry (GC-MS) analyses showed a significant biotransformation of heavy crude oil to light oil with 45.9% in aliphatic and 85.3% in aromatic fractions. Bhattacharya et al. [3], who used gas chromatography (GC) to elucidate the residuals of waste lubricating oil after biodegradation by *Ochrobactrum* sp. strain C1, found that benzene, naphthalene, azulene, indole, benzopyrene, and dibenzophenazine are predominant hydrocarbon structures. Robichaud et al. [41] reported that lightest (C_10_-C_16_) and heaviest (C_40_-C_50_) hydrocarbons were components of lubricant oil. After bioremediation, C_16_-C_22_ hydrocarbons were decreased at a rate of 63%, which was significantly more than those of hydrocarbons with other molecular sizes, including C_22_-C_28_, C_28_-C_34_, and C_34_-C_40_. This finding is consistent with that of Genov et al. [42], who observed that hydrocarbon-utilizing bacteria metabolically depleted low-molecular-weight hydrocarbons better than high-molecular-weight hydrocarbons.

Soil environments are unfavorable for inoculants to survive, establish niches, and become dominant species amongst indigenous microorganisms and predators [43]. To overcome this problem, we therefore evaluated the efficiency of immobilization materials, including agar and alginate, to prolong the survival and maintain the biodegradation activity of the inoculant after being introduced into the waste lubricating oil-contaminated soil environments. The result showed that the inoculation of the agar-immobilized cells of *Paenibacillus* sp. strain OL15 conferred the highest degradation ability (45%), followed by the alginate-immobilized cells (39%), and free cells (35%). The other species capable of degrading lubricant oil were previously reported. For example, Siddhi and Jinal [44] reported that after a 7-day cultivation of *Paenibacillus polymyxa* in a culture medium containing lubricant oil at concentrations of 1%, 5%, and 10%, its percentages of the degradation ability were 40%, 28%, and lower than 10%, respectively. *Pseudomonas* sp. and *Bacillus* sp. had the degradation ability of 63.46% and 2.76%, respectively, when cultivated in MSM containing 0.25% waste lubricating oil for 15 days [6]. Wang et al. [13] evaluated the degradation efficiencies of free cells, alginate-immobilized cells, and alginate-diatomite-immobilized cells of petroleum hydrocarbon degraders in the soils contaminated with 1% crude oil and found that, after a 20-day inoculation, alginate-diatomite-immobilized cells conferred the highest removal efficiency (29.8%), followed by alginate-immobilized cells (25.6%), and free cells (21.1%). Our study also found that agar was preferable to alginate as an immobilization material because it provided the higher number of surviving cells at the end of a 30-day storage. This finding is supported by our previous experiments. Nimnoi et al. [14] succeeded in using agar rather than alginate to prolong the survival time, support the niche establishment, and maintain the metabolic activity of the bacterial inoculant after being introduced into the complex soil environments. Consistently, Pongsilp and Nimnoi [15] also ascertained that agar was the most suitable immobilization material that supported the proliferation and promoted the metabolic activity of the potential inoculant, *Ensifer fredii* strain LP2/20, after being introduced into the field for 50 days. Our findings agree with previous studies which reported that immobilized cells showed a faster biodegradation rate than free cells, because immobilization materials protect bacterial cells from deleterious effects, enhance substrate bio-accessibility, and facilitate mass transport of oxygen and nutrients, which consequently increases the removal rates of contaminants [13,45,46,47,48].

In addition, the Illumina NGS results revealed that among all treatments, the phylum *Proteobacteria* was most abundant, followed by the *Acidobacteriota*, *Firmicutes*, and *Actinobacteriota*, respectively. Our result agrees with other reports which found the *Proteobacteria* to be the most dominant phylum in the lubricating oil-contaminated soils. For example, Meeboon et al. [49], who studied the difference in bacterial diversity of the lubricating oil-contaminated soil in the Songkla province, southern Thailand, found that members of the *Proteobacteria* were most abundant, ranging between 67.14 and 72.08%, followed by the *Actinobacteriota*, *Firmicutes*, and *Acidobacteriota*, respectively. The phyla *Proteobacteria*, *Firmicutes*, and *Actinobacteriota* were more abundant in the oil-contaminated soil collected from the Dagang oilfield, China [50]. The *Proteobacteria*, *Firmicutes*, and *Actinobacteriota* are widely used as the main biocatalysts for petroleum bioremediation because they synthesize many key enzymes, such as alkane monooxygenase, alcohol dehydrogenase, ring-hydroxylating dioxygenase, cytochrome P450, and aldehyde dehydrogenase that are necessary and involved in hydrocarbon degradation pathways [51,52].

However, any attempt at bioaugmentation, directly or indirectly, affects the remediation efficiency and microbial diversity in soil [51]. In this study, the inoculations of *Paenibacillus* sp. strain OL15 into the waste lubricating oil-contaminated soils resulted in the changes in bacterial community structure. The UPGMA analysis revealed that the relative abundance of bacterial phyla in inoculation treatments was different from that in an uninoculation treatment. An uninoculation treatment (treatment A) had the lowest bacterial diversity and richness when compared to inoculation treatments (treatments B, C, and D). The phylum *Proteobacteria* constituted the highest proportion in an uninoculation treatment, whereas its proportions were reduced in all inoculation treatments. The phylum *Firmicutes* became dominant in treatment B. The *Actinobacteriota* and *Campilobacterota* were increased in their proportions and became dominant phyla in treatments C and D, respectively. These shifts in dominant bacterial communities likely resulted from the different degrees of establishment of *Paenibacillus* sp. strain OL15 among treatments of the oil-contaminated soils. The abundance of *Paenibacillus* in treatments B, C, and D is in a decreasing order, which is directly proportional to the percentage of degradation. The high abundance of the phylum *Proteobacteria* in treatment A was mainly due to the high proportions of the genera *Pseudomonas*, *Vibrio*, *Herbaspirillum*, *Pseudoalteromonas*, *Massilia*, and *Duganella*. The high abundance of the phylum *Firmicutes* in treatment B was mainly due to the high proportions of the genera *Bacillus* and *Paenibacillus*. An increase in amount of the phylum *Actinobacteriota* in treatment C resulted from the presence of the dominant genus, *Gordonia*. The high proportion of the genus *Sulfurospirillum* resulted in the high abundance of the phylum *Campilobacterota* in treatment D. The shift in functional diversity between the uninoculated and inoculated soils possibly resulted from the change and enrichment of the bacterial community, in which the low metabolic activity was mainly due to the low amount of bacteria and the toxic effect of oil [11]. Jung et al. [51] reported the shift in dominant phylum from *Proteobacteria* to *Actinobacteriota* in the diesel-contaminated microcosms. Rahmeh et al. [52] revealed that the amounts of the *Proteobacteria*, *Chloroflexi*, *Chiorobi*, and *Acidobacteria* were increased during bioremediation of the oil-contaminated soil. Numerous soil factors, such as pH, OM, soil elements, nutrients, and hydrocarbon bioavailability, influenced the bacterial diversity and degradation rate [9,50]. Our result indicates that pH, OM, total N, P, Mg, Fe, and Zn were the major factors affecting the bacterial community composition. Peng et al. [53] demonstrated that OM, pH, N, and P were the factors affecting the variations in bacterial community composition in the oil-contaminated soils of Daqing oil field, China. The impact of soil pH, total N, P, K, and Zn on the shift in bacterial diversity in the petroleum-contaminated soils was also reported [50,54].

## 5. Conclusions

Bioaugmentation is already proven as one of the promising bioremediation methods for improving the degradation of waste lubricating oil in contaminated soil. *Paenibacillus* sp. strain OL15 manifested the highest ability to degrade waste lubricating oil in the contaminated soil environments. Using agar as an immobilization material better enabled the maintenance and promoted the survival of the inoculant than alginate. The inoculation of the agar-immobilized *Paenibacillus* sp. strain OL15 enhanced the degradation ability up to 6.4-fold over an uninoculated control. High-throughput sequencing of the 16S rRNA gene clarified that the *Proteobacteria*, *Acidobacteriota*, *Firmicutes*, *Actinobacteriota*, *Campilobacterota*, *Chloroflexi*, *Bacteroidota*, *Verrucomicrobiota*, *Desulfobacterota*, and *Myxococcota* were dominant phyla in the oil-contaminated soils. The inoculations of *Paenibacillus* sp. strain OL15 affected the soil bacterial community structures. The proportions of the genera *Gordonia*, MND1, *Azospira*, *Sphingomonas*, *Candidatus*, bacteriap25, *Nitrospora*, *Haliangium*, KD4-96, *Bryobacter*, *Rhodanobacter*, *Paucimonas*, *Sulfurospirillum*, *Clostridium*, *Citrifermentans*, Ellin6067, Subgroup_7, *Bacillus*, *Paenibacillus*, and JG30-KF-AS9 were altered in response to the establishment of *Paenibacillus* sp. strain OL15. Soil pH, EC, OM, total N, P, Mg, Fe, and Zn were the major factors influencing the bacterial community composition in the oil-contaminated soils. This study develops and implements an efficient bioremediation system employing the promising bioremediator and immobilization procedure for the degradation of waste lubricating oil in soil environments.

## Figures and Tables

**Table 1:** Percentage of degradation of waste lubricating oil by each bacterial isolate, as estimated in culture broth.

Isolate	Percentage of Degradation of Waste Lubricating Oil at a Concentration (v v^−1^) *			
	3	5	7	10
OL1	24.7 ± 0.7 ef ***	25.9 ± 0.4 f	7.7 ± 0 a	7.8 ± 1 b
OL2	38 ± 1 g	37 ± 3 h	10 ± 0.5 b	10.8 ± 0.3 c
OL3	9.9 ± 3 abc	bdl **	bdl	bdl
OL4	6.5 ± 1 ab	bdl	bdl	bdl
OL5	8.8 ± 2 abc	bdl	bdl	bdl
OL6	23 ± 2 ef	18 ± 0.7 d	10 ± 0.3 b	15.6 ± 0.6 d
OL7	26.3 ± 0.4 f	26.2 ± 0.2 f	10 ± 0.3 b	5.8 ± 0.6 a
OL8	36.8 ± 0.8 g	22.5 ± 2.2 e	15.6 ± 1 c	14.8 ± 1 d
OL9	10.7 ± 1 bc	bdl	bdl	bdl
OL10	5 ± 1 ab	bdl	bdl	bdl
OL11	13.5 ± 4.3 cd	6 ± 2.5 b	bdl	bdl
OL12	19.1 ± 3.6 de	5 ± 4.4 ab	bdl	bdl
OL13	45 ± 0.5 a	bdl	bdl	bdl
OL14	26.9 ± 1.7 f	13.7 ± 1.7 c	16 ± 1 c	10 ± 0.5 c
OL15	36 ± 0.9 g	32 ± 1.5 g	17.6 ± 0.5 d	15.5 ± 1 d

* All values are presented as means ± SD from triplicate samples. ** bdl, below detection limit. *** Values with the same letters in the column are not significantly different (*p* > 0.05) according to Tukey’s test.

**Table 2:** Number of surviving cells of free and immobilized cells with respect to strain, immobilization material, and incubation period.

Strain	Numbers of Surviving Cells from Immobilization Materials and Sterile Normal Saline at the End of Incubation Periods *					
	Agar (CFU g^−1^)		Alginate (CFU g^−1^)		Free Cells in Sterile Normal Saline (CFU mL^−1^)	
	15 Days	30 Days	15 Days	30 Days	15 Days	30 Days
*Enterobacter* sp. OL6	2 ± 0.2 × 10^6^ a **	2 ± 0.1 × 10^5^ a	7.9 ± 2.5 × 10^5^ a	5.3 ± 1.5 × 10^3^ a	4 ± 0.7 × 10^3^ a	5.2 ± 1 × 10^2^ a
*Klebsiella* sp. OL8	2.1 ± 0.2 × 10^8^ b	1.1 ± 0.3 × 10^6^ b	7.6 ± 1.4 × 10^5^ a	5.6 ± 1.8 × 10^4^ b	5 ± 0.2 × 10^4^ b	6.7 ± 0.7 × 10^2^ a
*Paenibacillus* sp. OL15	2.6 ± 0.3 × 10^8^ b	1.6 ± 0.3 × 10^7^ c	1 ± 0.1 × 10^6^ a	1.3 ± 0.2 × 10^5^ c	7 ± 1 × 10^4^ c	9 ± 0.7 × 10^2^ b

* All values are presented as means ± SD from triplicate samples. ** Values with the same letters in the column are not significantly different (*p* > 0.05) according to Tukey’s test. The initial cell concentration was 10^7^ cells g^−1^ immobilization material.

**Table 3:** Percentages of degradation of waste lubricating oil contaminated in the soil by the agar-immobilized, alginate-immobilized, and free cells of the strains at the end of a 30-day incubation period.

Strain	Percentage of Degradation of Waste Lubricating Oil Contaminated in the Soil *		
	Immobilization Materials		Cell Suspension
	Agar	Alginate	
*Enterobacter* sp. OL6	24 ± 7 cd **	14 ± 4 ab	13 ± 2 a
*Klebsiella* sp. OL8	28 ± 2 d	28 ± 2 d	19 ± 2 bc
*Paenibacillus* sp. OL15	45 ± 3 g	39 ± 1 f	35 ± 2 e

* All values are presented as means ± SD from triplicate samples. ** Values with the same letters are not significantly different (*p* > 0.05) according to Tukey’s test.

**Table 4:** Soil physicochemical properties from each treatment.

Parameter	Treatment			
	A	B	C	D
pH	7.33	6.85	6.73	6.97
Electrical conductivity (dS m^−1^)	1.7	2.8	2.8	2.1
Organic matter (g kg^−1^) *	1.15 ± 0 c **	0.64 ± 0 a	0.66 ± 0.02 a	0.8 ± 0 b
Total N (mg kg^−1^) *	36 ± 1 a	214 ± 7 c	207 ± 9 c	131 ± 3 b
Total P (mg kg^−1^) *	276 ± 15 a	346 ± 3 b	340 ± 7 b	339 ± 7 b
Total K (mg kg^−1^) *	555 ± 27 a	571 ± 26 a	547 ± 3 a	544 ± 5 a
Total Ca (mg kg^−1^) *	3919 ± 102 ab	3909 ± 30 ab	3750 ± 146 a	4018 ± 86 b
Total Mg (mg kg^−1^) *	891 ± 35 b	761 ± 11 a	760 ± 14 a	746 ± 27 a
Total Fe (mg kg^−1^) *	197 ± 7 c	47 ± 1 a	43 ± 3 a	154 ± 7 b
Total Zn (mg kg^−1^) *	45 ± 3 b	24 ± 0.6 a	23.7 ± 0.4 a	26 ± 1 a

* All values are presented as means ± SD from triplicate samples. ** Values with the same letters in the row are not significantly different (*p* > 0.05) according to Tukey’s test. Treatment A, an uninoculated control; B, inoculation with *Paenibacillus* sp. strain OL15 immobilized in agar; C, inoculation with *Paenibacillus* sp. strain OL15 immobilized in alginate; D, inoculation with a cell suspension of *Paenibacillus* sp. strain OL15.

**Table 5:** Bacterial diversity and richness of the waste lubricating oil-contaminated soil from each treatment.

Treatment	Number of Observed Species *	Diversity Indices *		Richness Indices *	
		Shannon–Weaver	Simpson	Chao1	ACE
A	2881 ± 299 a **	6.4 ± 0.4 a	0.9 ± 0 a	3196 ± 324 a	3272 ± 279 a
B	3497 ± 334 ab	7.5 ± 0.4 b	0.94 ± 0 b	3821 ± 382 ab	3877 ± 376 ab
C	3521 ± 60 b	7.5 ± 0.2 b	0.94 ± 0 b	3830 ± 83 ab	3879 ± 81 ab
D	3630 ± 157 b	7.7 ± 0.1 b	0.95 ± 0.01 b	3967 ± 146 b	4028 ± 156 b

* All values are presented as means ± SD from triplicate samples. ** Values with the same letters in the column are not significantly different (*p* > 0.05) according to Tukey’s test. Treatment A, an uninoculated control; B, inoculation with *Paenibacillus* sp. strain OL15 immobilized in agar; C, inoculation with *Paenibacillus* sp. strain OL15 immobilized in alginate; D, inoculation with a cell suspension of *Paenibacillus* sp. strain OL15.

**Table 6:** Top ten most abundant bacterial classes present in the waste lubricating oil-contaminated soil from each treatment.

Class *	Treatment			
	A	B	C	D
gamma-*Proteobacteria*	68 ± 5 b **	56 ± 4 a	54 ± 4 a	50 ± 2 a
*Acidobacteriae*	10 ± 1.27 a	9.6 ± 0.3 a	9 ± 0.7 a	9 ± 0.6 a
alpha-*Proteobacteria*	3.8 ± 0.4 a	5 ± 0.5 ab	5.5 ± 0.8 b	5 ± 0.5 ab
*Campylobacteria*	0.08 ± 0 a	1.2 ± 0.7 b	2 ± 0.1 b	4.6 ± 0.3 c
*Actinobacteria*	1 ± 0.36 a	2 ± 0.35 ab	3.35 ± 1.55 b	1.56 ± 0.2 ab
*Clostridia*	0.45 ± 0 a	1.92 ± 0.23 b	2 ± 0.12 b	3.7 ± 0.3 c
*Bacilli*	2 ± 0 a	2.74 ± 0.1 b	2 ± 0.15 a	2.2 ± 0.2 a
*Bacteroidia*	2 ± 0.15 a	2 ± 0.58 a	1.7 ± 0.2 a	2 ± 0.15 a
*Verrucomicrobiae*	1.2 ± 0.5 a	1.8 ± 0.2 ab	1.9 ± 0.5 ab	2.2 ± 0.3 b
*Desulfuromonadia*	0.2 ± 0 a	0.8 ± 0.1 b	1 ± 0.1 b	1.7 ± 0.1 c

* All values are presented as means ± SD from triplicate samples. ** Values with the same letters in the row are not significantly different (*p* > 0.05) according to Tukey’s test. Treatment A, an uninoculated control; B, inoculation with *Paenibacillus* sp. strain OL15 immobilized in agar; C, inoculation with *Paenibacillus* sp. strain OL15 immobilized in alginate; D, inoculation with a cell suspension of *Paenibacillus* sp. strain OL15.

## Data Availability

All data generated or analyzed during this study has been included in this published article. Sequence data has been deposited in the Sequence Read Archive of the NCBI under BioProject accession number PRJNA809146 and genomic accession numbers OM761074-OM761076.

## Supplementary Materials

The following supporting information can be downloaded at: https://www.mdpi.com/article/10.3390/biology11050727/s1, Figure S1: Neighbor-joining phylogenetic tree based on 16S rRNA gene sequences showing the taxonomic positions of the three selected strains among their neighbors; Figure S2: LEfSe analysis at multi taxonomic levels comparing the bacterial community compositions among treatments; Figure S3: CCA of bacterial classes and soil physicochemical characteristics; Table S1: Spearman’s (*r_s_*) correlations between abiotic and biotic factors.
