# Supplementary material for: Paenibacillus sp. Strain OL15 Immobilized in Agar as a Potential Bioremediator for Waste Lubricating Oil-Contaminated Soils and Insights into Soil Bacterial Communities Affected by Inoculations of the Strain and Environmental Factors
Source: Biology (Basel). 2022 May 9;11(5):727. doi: 10.3390/biology11050727 (PMC9138347; doi:10.3390/biology11050727)
Supplement: Supplementary file 1 [file biology-11-00727-s001.zip › biology-1686867-supplementary.pdf]

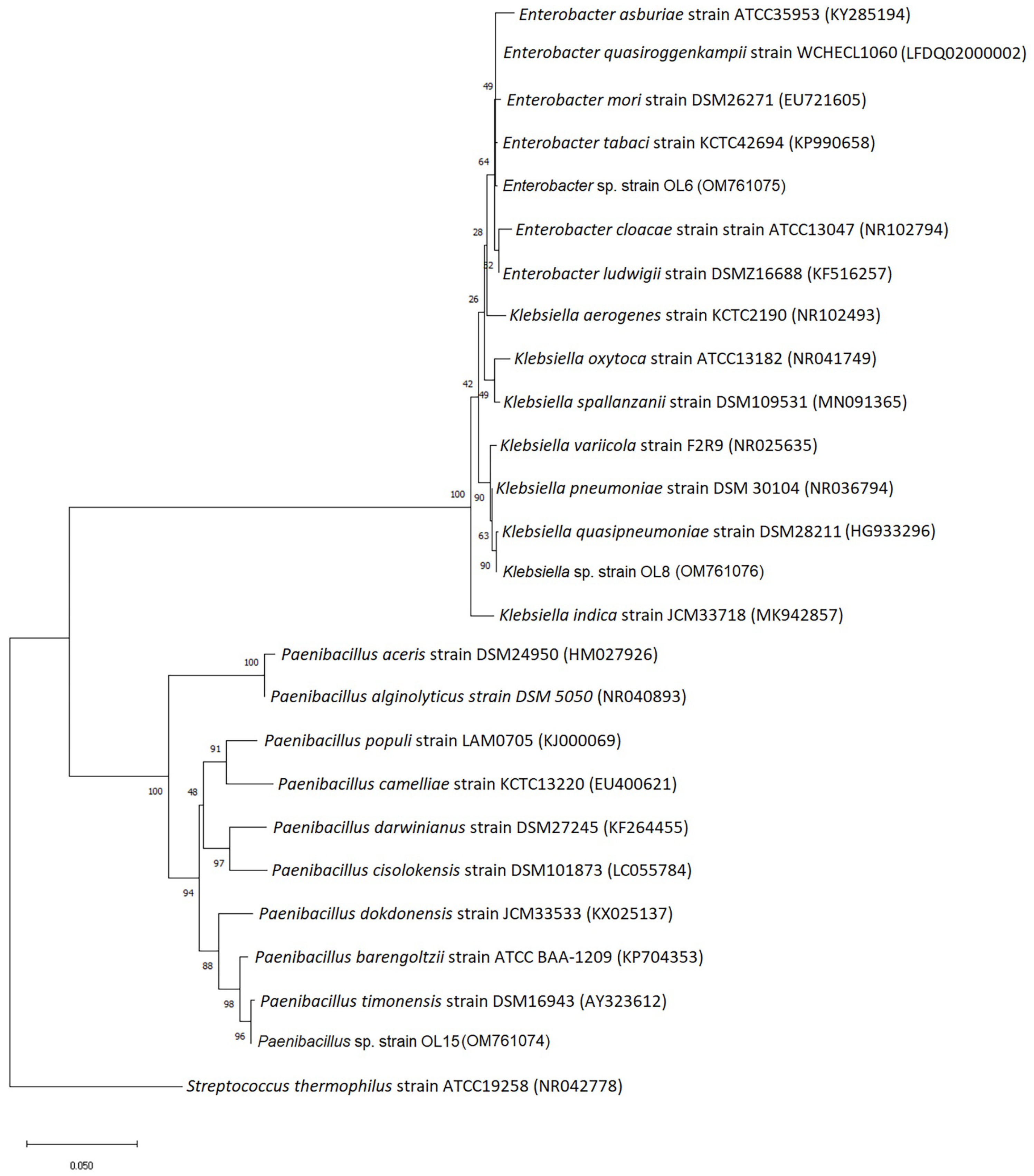

Supplementary Figure S1 Neighbor-joining phylogenetic tree based on 16S rRNA gene sequences showing the taxonomic positions of the three selected strains among their neighbors. The numbers at the nodes are bootstrap values based on re-sampling (1,000 replicates). NCBI accession numbers are in parentheses.

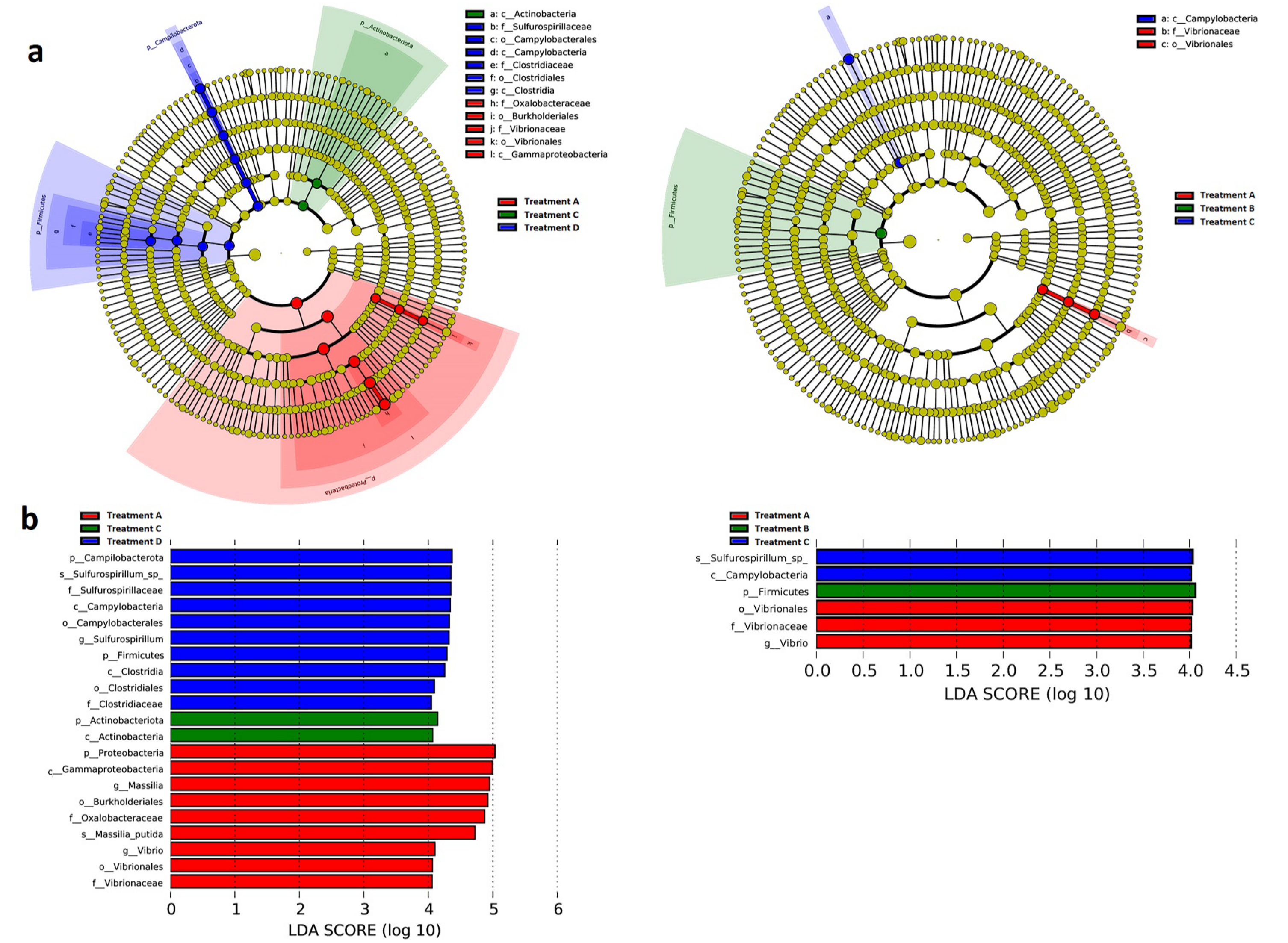

Supplementary Figure S2 LEfSe analysis at multi taxonomic levels comparing the bacterial community compositions among treatments. (a) Cladogram illustrating the most variable taxonomic groups in the bacterial community from each treatment. (b) Histogram of the LDA scores generated for the taxonomic groups which were differently abundant among the bacterial communities of the examined treatments. p, phylum; c, class; o, order; f, family; g, genus; s, species,

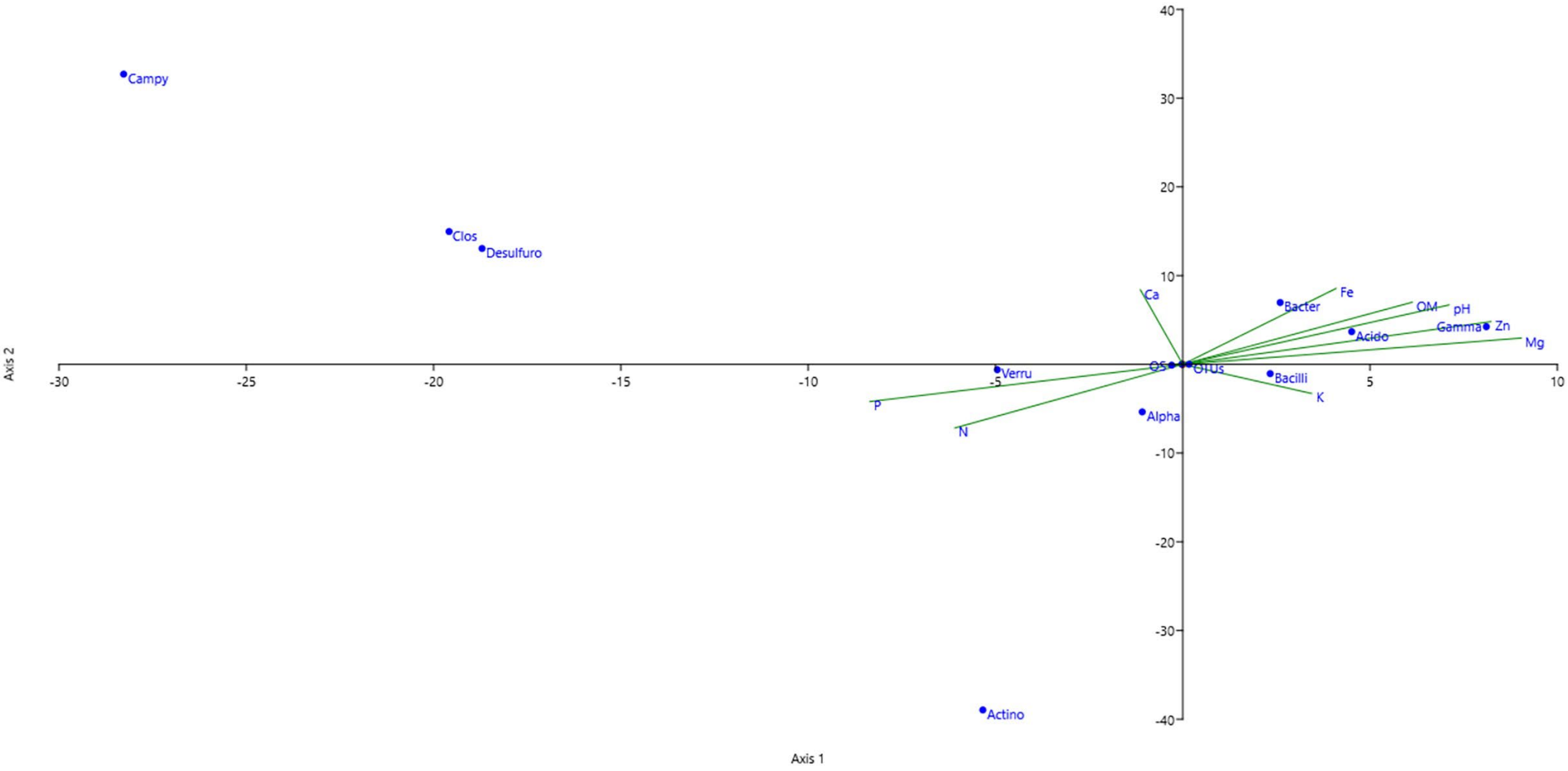

Supplementary Figure S3 CCA of bacterial classes and soil physicochemical characteristics. The influence of environmental factors on bacterial community structure is indicated by a green line. The length of each line represents the degree of the corresponding environmental factor on the distribution of bacterial class. Gamma, Gamma-*Proteobacteria*; Acido, *Acidobacteriae*; Alpha, Alpha-*Proteobacteria*; Campy, *Campylobacteria*; Actino, *Actinobacteria*; Clos, *Clostridia*; Bacter, *Bacteroidia*; Verru, *Verrucomicrobiae*; Desulfuro, *Desulfuromonadia*.

Supplementary Table S1 Spearman's (r_s) correlations between abiotic and biotic factors

Abiotic factors	Spearman's (r_s) correlations between abiotic and biotic factors											
	Biotic factors*											
	Gamma-*Proteobacteria*	*Acidobacteriae*	Alpha-*Proteobacteria*	*Campylobacteria*	*Actinobacteria*	*Clostridia*	*Bacilli*	*Bacteroidia*	*Verrucomicrobiae*	*Desulfuromonadia*	OTUs	Observed species
pH	0.34 (0.27)	0.32 (0.30)	-0.66 (0.01)**	-0.38 (0.21)	-0.79 (0.00)**	-0.36 (0.24)	-0.10 (0.73)	0.34 ()	-0.49 (0.27)	-0.38 (0.10)	-0.43 (0.16)	-0.38 (0.21)
Ec	-0.23 (0.45)	-0.17 (0.59)	0.49 (0.10)	0.19 (0.54)	0.75 (0.00)**	0.21 (0.50)	0.56 (0.05)	-0.08 (0.79)	0.34 (0.27)	0.19 (0.54)	0.49 (0.10)	0.47 (0.11)
OM	0.26 (0.40)	0.11 (0.71)	-0.44 (0.14)	-0.19 (0.54)	-0.76 (0.00)**	-0.23 (0.47)	-0.54 (0.06)	0.04 (0.89)	-0.39 (0.19)	-0.21 (0.51)	-0.49 (0.10)	-0.49 (0.10)
TotalN	-0.22 (0.49)	-0.21 (0.50)	0.56 (0.05)	0.23 (0.46)	0.68 (0.01)**	0.20 (0.51)	0.39 (0.20)	-0.13 (0.68)	0.25 (0.41)	0.20 (0.51)	0.51 (0.08)	0.46 (0.12)
TotalP	-0.38 (0.21)	-0.37 (0.22)	0.67 (0.01)**	0.35 (0.26)	0.74 (0.00)**	0.37 (0.23)	0.59 (0.04)**	0.19 (0.54)	0.44 (0.15)	0.33 (0.28)	0.69 (0.02)**	0.62 (0.01)**
TotalK	0.12 (0.69)	-0.43 (0.15)	0.25 (0.41)	-0.07 (0.82)	0.16 (0.60)	-0.02 (0.94)	0.02 (0.93)	-0.18 (0.55)	-0.06 (0.84)	-0.15 (0.63)	0.14 (0.66)	0.13 (0.68)
TotalCa	-0.37 (0.22)	0.39 (.20)	-0.32 (0.29)	0.29 (0.35)	-0.10 (0.74)	0.21 (0.49)	0.16 (0.61)	0.48 (0.11)	0.21 (0.51)	0.25 (0.41)	0.14 (0.66)	0.21 (0.51)
TotalMg	0.73 (0.00)**	0.21 (0.51)	-0.35 (0.25)	-0.63 (0.02)**	-0.70 (0.01)**	-0.59 (0.04)**	-0.14 (0.64)	-0.16 (0.60)	-0.54 (0.06)	-0.64 (0.02)**	-0.67 (0.01)**	-0.67 (0.00)**
TotalFe	0.30 (0.34)	0.10 (0.74)	-0.44 (0.15)	-0.28 (0.36)	-0.78 (0.00)**	-0.29 (0.35)	-0.27 (0.39)	0.18 (0.55)	-0.43 (0.15)	-0.25 (0.43)	-0.42 (0.16)	-0.45 (0.13)
TotalZn	0.21 (0.49)	0.20 (0.52)	-0.67(0.01)**	-0.30 (0.34)	-0.63 (0.02)**	-0.28 (0.37)	-0.28 (0.37)	0.37 (0.23)	-0.36 (0.24)	-0.29 (0.35)	-0.32 (0.29)	-0.26 (0.40)

*Data is shown in format of r_s (*P*-value).

**Correlation is significant at the 0.05 level.
